# PET and SPECT Imaging of the EGFR Family (RTK Class I) in Oncology

**DOI:** 10.3390/ijms22073663

**Published:** 2021-04-01

**Authors:** Sara S. Rinne, Anna Orlova, Vladimir Tolmachev

**Affiliations:** 1 Department of Medicinal Chemistry, Uppsala University, 751 23 Uppsala, Sweden; sara.rinne@ilk.uu.se (S.S.R.); anna.orlova@ilk.uu.se (A.O.); 2 Science for Life Laboratory, Uppsala University, 752 37 Uppsala, Sweden; 3 Research Centrum for Oncotheranostics, Research School of Chemistry and Applied Biomedical Sciences, Tomsk Polytechnic University, 634050 Tomsk, Russia; 4 Department of Immunology, Genetics and Pathology, Uppsala University, 752 37 Uppsala, Sweden

**Keywords:** molecular imaging, PET, SPECT, RTK Class I, EGFR, HER1, HER2, HER3, HER4

## Abstract

The human epidermal growth factor receptor family (EGFR-family, other designations: HER family, RTK Class I) is strongly linked to oncogenic transformation. Its members are frequently overexpressed in cancer and have become attractive targets for cancer therapy. To ensure effective patient care, potential responders to HER-targeted therapy need to be identified. Radionuclide molecular imaging can be a key asset for the detection of overexpression of EGFR-family members. It meets the need for repeatable whole-body assessment of the molecular disease profile, solving problems of heterogeneity and expression alterations over time. Tracer development is a multifactorial process. The optimal tracer design depends on the application and the particular challenges of the molecular target (target expression in tumors, endogenous expression in healthy tissue, accessibility). We have herein summarized the recent preclinical and clinical data on agents for Positron Emission Tomography (PET) and Single Photon Emission Tomography (SPECT) imaging of EGFR-family receptors in oncology. Antibody-based tracers are still extensively investigated. However, their dominance starts to be challenged by a number of tracers based on different classes of targeting proteins. Among these, engineered scaffold proteins (ESP) and single domain antibodies (sdAb) show highly encouraging results in clinical studies marking a noticeable trend towards the use of smaller sized agents for HER imaging.

## 1. Introduction

### 1.1. Human Epidermal Growth Factor Receptor Family in Cancer

The human epidermal growth factor receptor (EGFR) family (also designated receptor tyrosine kinase (RTK) Class I, ErbB family or HER-family) is a class of tyrosine kinase receptors involved in fundamental cellular processes such as cell proliferation, migration, survival, and angiogenesis [1,2]. The four family members, HER1 (also ErbB1 or EGFR), HER2 (ErbB2), HER3 (ErbB3), and HER4 (ErbB4) have a common structure consisting of an extracellular domain (ECD), a transmembrane domain, and an intracellular domain (ICD) with a tyrosine kinase and a c-terminal tail [1,2]. The ECD consists of four subdomains, of which domain I and III are leucine-rich and involved in ligand binding. Domain II and IV are cysteine-rich and involved in intramolecular interaction [1]. With the exception of HER2, the receptors normally exist in a non-activated monomeric state with conformation restricted by an intramolecular tether between subdomain I and III [3,4]. Ligand binding causes transformation of the receptor’s extracellular conformation into an activated state. Domains I and III form a ligand binding pocket and expose subdomain II to enable dimerization with other family members [3,4,5,6,7,8]. HER2 exists in a steady opened conformation allowing for dimerization without binding to a ligand [1]. Dimerization can occur between identical receptors (homodimerization), e.g., HER1-HER1, or with another family member (heterodimerization), e.g., HER2-HER3. Ligand binding and dimerization triggers biochemical downstream signaling by inducing kinase activity and phosphorylation of tyrosine residues on the intracellular c-terminal tail of the receptors [9,10]. HER3 lacks sufficient intracellular kinase activity and its signaling is, therefore, reliant on heterodimerization with other family members [11,12]. Several natural ligands interact with members of EGFR family members. Epidermal growth factor (EGF), transformation growth factor alpha (TGF-α), amphiregulin, and epiregulin are HER1 specific ligands. Heparin-binding EGF-like growth factor (HB-EGF), betacellulin, and epigen bind both HER1 and HER4. The neuregulins (NRG1-4) are natural ligands binding to HER3 and HER4. There are no known natural ligands binding HER2 with high affinity [13,14,15,16]. The EGFR signaling network is an intricate, tightly knit system with well-balanced interactions [1,12,17]. Common pathways activated by EGFR-family members include the phosphatidylinositol3-kinase (PI3K)/Akt pathway (mediating, among other things, cell survival) and the Ras/Raf/MEK/ERK1/2 and phospholipase C (PLCγ) pathways (mediating cell proliferation) [18,19].

Overexpression of the receptors of the EGFR family, functional alterations and deregulation of downstream signaling have been closely linked with oncogenesis and disease progression [12,17,20]. HER1 and HER2 have been implicated in oncogenic transformation as early as in the 1980s [21,22,23]. HER1 is overexpressed in non-small cell lung cancer (NSCLC), advanced prostate cancer (PC), head and neck cancer, and colon and pancreatic cancer [24]. HER2 overexpression is most prominently associated with its role in breast cancer (BC), where it is over expressed in 15–20% of cases [23,25]. It is also overexpressed in other malignancies, such as ovarian, gastric, prostate and pancreatic cancer [26,27,28]. HER3 was discovered later and the level of HER3 overexpression in cancer is relatively low compared with HER1 and HER2 [29,30]. HER3 overexpression has for example been associated with breast, ovarian, gastric, and prostate cancer [31,32,33,34,35] and linked to poor survival [33,36]. It has been shown that HER3 expression can be upregulated in response to HER1 or HER2 targeted therapies to compensate for proliferative signaling loss by activation of the PI3K/Akt pathway [37,38,39]. HER4 is the least explored receptor of the EGFR family and its role and mechanisms of signaling are still poorly understood. There are indications that HER4-signaling can result in both a pro-tumor and anti-tumor effect [16,40,41]. In colorectal cancer, HER4 expression seems to be implicated in disease progression [42,43]. In breast cancer, HER4 expression has been reported as a marker for improved outcome [44,45] but also as a marker of trastuzumab resistance [46] and decreased sensitivity to tamoxifen [47]. 

The prominence and documented involvement of EGFR-family receptors in tumorigenesis makes them highly relevant candidates for targeted therapy. The development of therapeutic agents continues to advance with more and more agents on their way into clinical practice. Small molecule tyrosine kinase inhibitors (TKI) can be used to suppress the intracellular kinase activity of EGFR-family receptors. Selective inhibition of HER-function using monoclonal antibodies (mAbs) or other molecules binding to the receptors’ extracellular domain is another strategy [48,49,50,51]. Cetuximab (Erbitux) is an HER1-targeting mAb approved for metastatic colorectal cancer and non-small cell lung cancer, and head and neck cancer. Panitumumab (Vectibix) is another anti-HER1 mAb used for treatment of patients with colorectal cancer. Trastuzumab (Herceptin) and pertuzumab (Perjeta^®^) are mAbs targeting different epitopes of the HER2 that are routinely used for the treatment of breast cancer patients. No therapeutic agents targeting HER3 are currently approved for clinical use, but HER3 targeting mAbs AMG-888 (patritumab) and lumretuzumab (RG7116) are undergoing clinical trials. Because the role of HER4 in cancer is still poorly understood, the development of targeting therapeutics is lacking.

### 1.2. Detection of EGFR-Family Expression for Targeted Treatment

Therapeutic approaches selectively targeting the EGFR family of receptors require that patients with HER expression who could benefit from these therapies are identified beforehand. Furthermore, methods are required to follow the expression status over time to detect potential changes in the receptors’ expression during disease progression and in response to treatment.

### 1.3. Biopsy Based Methods

Biopsy based methods are the most commonly used to detect the presence of EGFR-family members. However, invasiveness of the procedure is a major drawback. Furthermore, the accuracy of biopsies can be compromised by intra- and inter-tumor heterogeneity causing false-negative results [52]. Inter-tumor heterogeneity essentially requires sampling from all metastases, which might not be possible with biopsy-based methods if there are many tumor sites or if the accessibility is limited. In addition, HER1, HER2 and HER3 are known to have discordant expression levels between primary tumors and metastases, and to change expression during tumor development and therapy [53,54,55,56]. Thus, more comprehensive, alternative methods for regular whole-body monitoring of HER-expression status are needed to improve patient management for HER-targeted therapy.

### 1.4. Radionuclide-Based Molecular Imaging

Positron Emission Tomography (PET) and Single Photon Emission Tomography (SPECT) are radionuclide-based molecular imaging modalities. In contrast to biopsy-based methods, they can repeatedly and non-invasively visualize target expression. Hence, they are well suited for patient selection for HER-targeted treatment, monitoring of treatment response and changes in target expression, as well as optimization of dosing for therapy. In addition, PET and SPECT can detect expression in the whole body within one procedure, reducing the risk of false-negative results due to intra-tumoral heterogeneity [52]. The importance of methods for detection of receptors expression other than biopsy was later convincingly illustrated. Bensch et al. performed a study on the influence of a [^89^Zr]Zr-trastuzumab PET image on diagnostic understanding and clinical decision-making for patients with HER2 positive breast cancer [57]. In this study, treatment-decisions were altered in 40% of the patients based on results from the PET-imaging. [^89^Zr]Zr-trastuzumab-PET scans also increased physicians’ confidence in the existing treatment plan in 50% of the cases and improved physicians’ understanding of the disease in almost 90% of cases. Both PET and SPECT can be combined with MRI or CT for additional anatomical information, though combination with CT is more common than MRI. SPECT cameras are widely available and cheaper than PET-cameras, but PET offers better sensitivity and quantification accuracy [58,59].

Different radionuclides are used for PET and SPECT imaging [60]. SPECT-imaging is mainly performed using ^99m^Tc, ^123^I, and ^111^In, which are commercially available for clinical use [61]. Technetium can be produced in house via a ^99^Mo/^99m^Tc-generator. ^123^I and ^111^In can be shipped from the production site due to the convenience of their longer half-lives. Common clinically used PET nuclides are ^11^C, ^18^F, ^68^Ga [62]. The rather short half-life of these nuclides limits their application to tracers with fast pharmacokinetics. ^55^Co, ^64^Cu, and ^89^Zr are suitable radiometals for PET-imaging, offering a longer half-life which is much needed for targeting molecules with long circulation times, e.g., mAbs. GMP-grade production and worldwide distribution of ^89^Zr has been established, and production of both ^55^Co and ^64^Cu has been shown to be feasible on medical cyclotrons.

[^18^F]FDG is the most used PET-tracer in clinical oncology. As a glucose analog, it can detect abnormalities in glucose metabolism, which is characteristic for many types of cancer. Clinically, [^18^F]FDG is utilized, for example, for cancer staging, detecting tumor recurrence, and for pre-surgery or radiotherapy planning [63,64]. SUV-values from [^18^F]FDG-PET are a surrogate marker for lesion malignancy and treatment response. They do not, however, give information about the expression of potential therapeutic molecular targets, such as EGFR-family members on the cell surface of cancer cells [65]. Currently, as precision medicine is evolving, simple detection of tumors is no longer sufficient and diagnostic PET and SPECT tracers that can selectively image therapeutic targets are needed. Nonetheless, the HER-targeting imaging agents that will be discussed in this review are not necessarily designed as primary diagnostic agents for malignant lesions (expression of the potential targets is not guaranteed in all malignant sites, due the proven inter-patient and inter tumor-heterogeneity). These tracers are rather intended to complement the existing diagnostic test, aid decision-making and make patient care more efficient.

### 1.5. General Considerations for the Development of Radiotracers for PET and SPECT Imaging

Tracer design should consider the type of the targeting molecule, radionuclide (physical half-life matching the biological half-life of the targeting molecule) and labeling chemistry, affinity to the target, receptor expression, retention of the tracer in the target tissue, and off-target interactions with normal tissues.

A diagnostic radiotracer should provide high sensitivity and specificity, and the tumor uptake of the tracer should correlate to the receptor expression level. The sensitivity of a scan is directly related to the imaging contrast, meaning the ratio between tracer uptake in the tumor(s) and normal tissues, particular in the vicinity of the tumor(s). The imaging contrast can be reduced appreciably by activity circulating in the blood. An efficient clearance of the tracer from the blood is therefore desirable. The most probable metastatic sites depend on the medical context. The tracer should provide high tumor-to-non-tumor ratios for these tissues. For example, the liver, lungs and bones are common metastatic sites in breast cancer [66]. Thus, high tumor-to-liver, tumor-to-lung and tumor-to-bone ratios are essential for detection of HER2-positive metastatic breast cancer. Natural receptor expression in normal tissues, off-target interactions of the tracer with non-targeted tissue, and clearance pathways can limit the tumor-to-background ratios. This is an apparent problem for the detection of HER1 or HER3 expressing hepatic tumors and metastases due to the endogenous expression of these receptors in the liver. Adjusting the dose with a non-labeled molecule to occupy the natural receptors in the liver might be a way to improve the tumor-to-liver ratio and has, for example, been applied for imaging of HER1 expression [67].

Detection of small tumors and metastases is challenging due to the partial volume effect (PVE), causing the underestimation of activity in small lesions. This increases the need for high-tumor to non-tumor ratios to provide sufficient sensitivity [68,69]. For example, a tumor-to-background ratio of 16–18 is needed to detect a metastasis with a size of 5 mm, whereas a ratio of roughly 90 is required to detect a metastasis with a size of 3 mm [70].

Currently, a variety of targeting molecules of different sizes and origins are explored for imaging of EGFR-family members. Radiolabeling of existing therapeutic mAbs for PET or SPECT has pre-dominated the development of imaging agents. Antibody-based tracers have several drawbacks and tracers of smaller size are emerging as serious competitors for the imaging of EGFR-family receptors and other targets [71,72]. The long circulation time and slow extravasation of antibodies contributes to substantial background from the blood and shifts the optimal imaging time point to 3–6 d post injection (pi) [73,74,75]. The pharmacokinetics and blood clearance of a potential tracer is, to a large extent, dependent on their size. The capillary permeability decreases with the increasing molecular weight and effective molecular radius of the protein [76]. Thus, a smaller imaging agent can provide better extravasation, deeper penetration into the tumors, and rapider clearance of the non-bound agent from circulation and may enable high imaging contrast a few hours after injection [71,77,78,79,80]. The physiological uptake of antibodies in the liver and hepatobiliary excretion are additional problems with antibody-based tracers that limit the detection of potential metastases [71,73,81]. For proteins with a molecular weight less than 60 kDa, the primary elimination pathway is generally renal [77,82,83]. The enhanced permeability and retention effect (EPR effect) contributes to unspecific accumulation of radiolabeled antibodies in the tumor and reduces the imaging specificity [84,85].

Antibody fragments (e.g., F(ab′)_2_, Fab), engineered single chain variable fragments (scFvs), single domain antibodies (sdAb, also known as nanobodies) and engineered scaffold proteins (ESPs), e.g., affibody molecules, designed ankyrin repeat proteins (DARPins), and ABD Derived Affinity Proteins (ADAPTs), have shown to be promising targeting agents for molecular imaging [78,86,87,88,89,90,91].

Fab or F(ab′)_2_ antibody fragments (Mw of ~55 kDa and ~110 kDa, respectively) are produced by degradation of the Fc-region via enzymes pepsin and papain [92]. Their smaller size overall increases their clearance from the blood, and enables reasonable imaging contrast earlier than in the case of intact antibodies [71]. Despite the lower molecular weight, the effective molecular radius of F(ab′)_2_ fragments is only slightly smaller than full-length antibodies (5.05 nm vs. 5.5 nm), which still limits their extravasation compared with other smaller targeting molecules [76]. scFvs, which consist of the variable domains of light and heavy chains (VL and VH) linked by a polypeptide linker, have an even smaller size than antibody fragments (25–27 kDa). They often reach peak uptake in tumors within hours after injection. Dimers ((scFv)_2_) or other derivatives of scFvs (diabodies, minibodies) are also suitable as imaging probes. Single domain antibodies (sdAb or nanobodies) are even smaller than scFvs as they only consist of mAb heavy chains [92].

Engineered scaffold proteins (ESPs) contain a robust protein framework tolerating temperatures of up to 95 °C, high molarity buffers, and a wide range of pH. Affibody molecules, ADAPTs and DARPins are designed based on different scaffolds, but all have a molecular weight in the range of 4–20 kDa [71] and can be selected against different targets, including receptors of the EGFR-family. The three-helical affibody molecules are derived from the B-domain of staphylococcal protein A, with a molecular weight of around 7 kDa [93]. ADAPTS are based on the albumin binding domain of protein G and have a molecular weight of around 5.2 kDa [94]. The DARPin scaffold contains four or five repeats, each having two anti-parallel alpha-helices and a beta-turn; it is slightly bigger than affibody molecules and ADAPTS (12 to 18 kDa) [95]. A significant advantage of ESPs is the flexibility of their design. Small modifications in the scaffold amino acid composition, the addition of hydrophilic tags and linkers, or the use of different chelators and nuclides usually do not drastically influence the ESPs’ affinity to the target. However, they can have an appreciable effect on their biodistribution and have shown to be an important tool for improving the imaging contrast of EGFR-family targeting ESPs [86].

Small peptides have the same features as ESPs [96,97], but the risk of negatively impacting the binding is much higher. Finally, small-molecule TKI are another class of potential targeting molecules that have been evaluated for the imaging of receptors of the EGFR-family [98]. However, TKIs usually target the intracellular kinase rather than a specific receptor, which results in completely different requirements. Therefore, TKI are not further included in this review.

Regardless of the choice of the targeting molecule, the half-life of the selected radionuclide should match the biological half-life of the tracer to minimize the radiation dose to the patient. The radionuclide needs to be stably attached to the targeting molecule to prevent the release and re-distribution of the free nuclide because this could negatively affect the imaging contrast and, therefore, the sensitivity. Labeling chemistry should be efficient: minimize handling of the radionuclide, take into account the nuclide half-life, and provide high labeling yield and purity of the product [99]. Non-metallic radionuclides (e.g., ^18^F, ^11^C) are generally incorporated into the targeting agent through the formation of a covalent bond, whereas radiometals (e.g., ^68^Ga, ^111^In, ^64^Cu, ^89^Zr) are attached to the targeting molecule via chelation using bifunctional chelators (BFC) [99].

The internalization of radiolabeled probes into targeted cells and intracellular retention of the radionuclide after the probe’s degradation is another factor for consideration when evaluating potential imaging agents [100,101]. After binding of the targeting probe, the receptor-ligand complex can be internalized. For example, EGF-stimulated/activated internalization of HER1 is approximately 10-fold faster than for the monomeric, non-activated receptor [102]. The radiometabolites of so-called residualizing labels are retained or “trapped” in the cells after internalization and degradation of the tracer by proteolytic enzymes in the lysosomes. These are labels with hydrophilic (polar or charged) radiometabolites, often radiometals, e.g., ^111^In, ^68^Ga. Lipophilic radiometabolites can diffuse through the cell membrane and leak out of the cells after internalization of the tracer. These labels are called non-residualizing labels and are typically radiohalogens, e.g., iodine. Residualizing labels are preferred for rapidly internalizing probes, to ensure retention of activity in the tumors. It is important to note that the residualizing properties also apply to non-targeted tissues where a probe can be internalized (e.g., kidneys and liver). High activity retention in these tissues could, therefore, impact the imaging contrast. With slow internalizing probes (that do not trigger internalization when bound to the receptor), the type of label is less crucial, but affinity should be high to ensure retention of the radiolabeled tracers bound to the receptors on the cell surface. ^89^Zr, followed by ^64^Cu and ^111^In are the most popular nuclides for the labeling of mAbs, due to their longer half-lives (3.3 d, 12.7 h, 2.8 d respectively). For the smaller targeting molecules, the list of potential radiolabels increases, due to their faster kinetics and better penetration, allowing for more freedom in finding the most optimal match for the targeting molecule, labeling procedure and application.

High affinity of the radiotracer is particularly important for good imaging contrast when imaging low expressing targets, e.g., HER3 [103]. Recently developed ESPs binding HER1, HER2 and HER3 have shown affinities in the sub-nanomolar range and demonstrated promising data in preclinical imaging of these targets [88,89,104,105,106,107].

## 2. This Review

This review focuses on the recent progress in PET and SPECT imaging of EGFR-family receptors HER1, HER2, HER3 using different types of targeting agents. Due to its ambiguous stance and the lack of HER4-targeting therapeutic and diagnostic agents, PET and SPECT imaging of HER4 will not be discussed here. Even though the receptors of the EGFR-family are closely related in their structure and function, as a target for molecular imaging each of them come with individual challenges and requirements to potential radiotracers. Each receptor will be discussed in a separate section, touching upon its individual challenges as an imaging target, current status, and preclinical and clinical advances in tracer development. We hope to create a general overview for PET and SPECT imaging of receptors of EGFR-family members in oncology.

## 3. HER1 Imaging

The appreciable natural expression of HER1 in healthy tissue is one of the major challenges for high image contrast. One of the main problematic organs is the liver, which is very well perfused and presents with a substantial HER1 expression on hepatocytes [22,108].

Radiolabeled variants of EGF-based ligands and therapeutic anti-HER1 mAbs were the first targeting molecules considered for the imaging of HER1 expression. However, EGF-based ligands are not ideal for clinical application because the adverse effects (nausea, vomiting, hypotension, fever, and chills) caused by its agonistic action lead to substantial discomfort for the patients [109]. Furthermore, a direct comparison of [^111^In]In-DTPA-hEGF with the anti-HER1 monoclonal antibody MAb 528 demonstrated much lower tumor uptake, surprisingly higher liver uptake, and notably lower tumor-to-tissue ratios for [^111^In]In-DTPA-hEGF [110]. This suggested the inferiority of small EGF-based ligands to antibody-based tracers for the imaging of HER1. The use of radiolabeled mAbs have proceeded furthest in the development of HER1 targeting imaging agents, but none of the tracers have been approved for clinical use yet.

A factor that should be considered when comparing different HER1 targeting agents in preclinical studies is the issue of cross-reactivity to both human and murine HER1. In contrast to engineered scaffolds, such as affibody molecules, most antibodies or antibody-derived fragments do not bind the murine analog of HER1. This means that the interaction of the tracers with receptors expressed in healthy organs are difficult to model, which should be kept in mind when interpreting results.

### 3.1. Monoclonal Antibodies for Imaging of HER1 Expression

**Figure 1:**
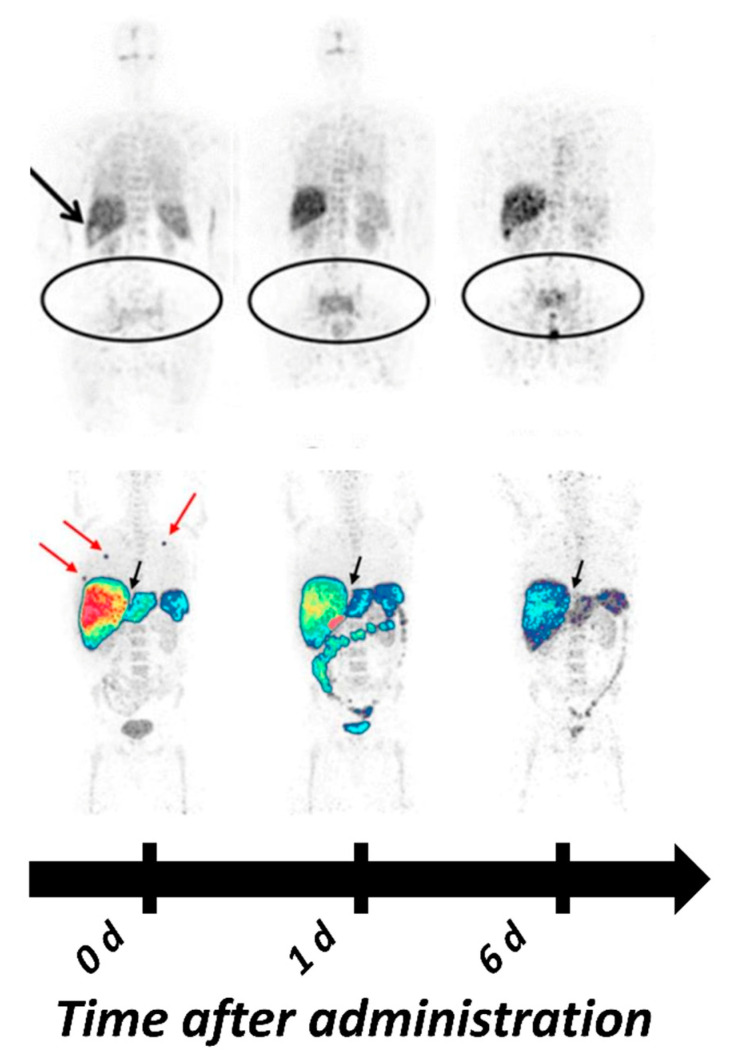
Imaging of HER1 expression in patients using ^89^Zr-labeled monoclonal antibodies cetuximab (upper row) [75] and panitumumab (bottom row) [81]. Photopenic lesions in liver are indicated with black arrows; accumulation in lungs pointed with red arrows, resolved with time. These images were originally published in Oncotarget [75] and Am J Nucl Med Mol Imaging [81].

The therapeutic mAb cetuximab labeled with ^89^Zr is the tracer that is the most advanced in (clinical) development for the imaging of HER1 expression. Recent studies have demonstrated the absence of adverse effects [111] and specific tracer uptake with optimal imaging 6–7 days post injection (pi) [75,111,112]. Examples of [^89^Zr]Zr-cetuximab-based PET-imaging in clinics are presented in Figure 1. Even et al. reported a significant difference in the SUVmax between patients with high and low HER1 expression and in patients with advanced head and neck cancer, but also a large variation in tumor-to-background ratios between patients. Interestingly, the highest tumor-to-background ratio belonged to the patient with the lowest HER1 expression, according to immunohistochemistry (IHC) [112]. In a recent review van Dijk with co-authors have summarized factors influencing the HER1 imaging predictive value in solid tumors: HER1 could be bypassed by other HER and G-protein-coupled receptors pathways, mutations in the downstream signaling cascade of the HER1 and the tumor suppressor protein p53 [113].

As an alternative to the ^89^Zr label, [^64^Cu]Cu-DOTA-cetuximab has been characterized in preclinical studies showing satisfactory tumor uptake and clear HER1 visualization in a panel of xenografts (A431, TE-8, TE4, UM-SCC-22B, SCC1) [114,115,116]. In esophageal cancer xenografts, the uptake of [^64^Cu]Cu-DOTA-cetuximab increased steadily and peaked at 48 h [116]. As expected, the liver is the normal organ with the highest activity uptake. However, with either tracer, tumor-to-blood ratios did not exceed 10 in all tested models [114,115,116].

Similar to cetuximab, the antibody panitumumab has a sub-nanomolar affinity towards HER1. Direct comparison of ^86^Y-labeled CHX-A”-DTPA-cetuximab and [^86^Y]Y-CHX-A”-DTPA-panitumumab in a malignant mesothelioma model suggested the superiority of [^86^Y]Y-CHX-A”-DTPA-panitumumab for PET-imaging of HER1 expression due to a slightly higher tumor uptake and significantly lower liver uptake [117,118]. Lindenberg et al. in 2017 reported the first human experience of [^89^Zr]Zr-panitumumab imaging in three patients with metastatic colorectal cancer, showing the safety of the tracer (Figure 1) [81]. However, no tumors could be visualized, which might be due to their location in the liver and abdomen, areas with high metabolic accumulation of [^89^Zr]Zr-panitumumab. In addition, n o unlabeled compound was co-injected to potentially reduce HER1-mediated hepatic uptake. So-called “cold loading”, meaning the spiking of the injection mixture with a non-labeled protein to saturate HER1 receptors in the liver and increase the concentration of the radiolabeled imaging agent in the blood circulation, has been shown to be a feasible approach in clinical and preclinical studies [119,120]. Currently, two clinical trials with [^89^Zr]Zr-panitumumab are ongoing (clinical trials.gov, NCT03764137, NCT03733210).

In preclinical studies, panitumumab has also been labeled with ^111^In for SPECT imaging. [^111^In]In-CHX”-DTPA-panitumumab showed a similar biodistribution to [^89^Zr]Zr-DFO-panitumumab, but slightly faster blood clearance and lower uptake in the lung [121]. In an attempt to reduce accumulation of ^111^In-labeled panitumumab in the liver and spleen, panitumumab was conjugated to a metal-chelating polymer (MCP) with a polyglutamide backbone containing an average of 13 DOTA chelators [122]. The modified panitumumab conjugate ([^111^In]In-panitumumab-MCP) provided an uptake similar to [^111^In]In-DOTA-panitumumab in PANC-1 xenografts and was able to clearly visualize them using SPECT. Surprisingly, the hepatic uptake was significantly higher for [^111^In]In-panitumumab-MCP (2.9 fold) than for the control [122].

Nimotuzumab is a third full-length mAb investigated as a HER1 imaging agent. In preclinical studies, the tumor uptake of [^89^Zr]Zr-DFO-nimotuzumab increased up to 168 h pi, but HER1 expression in breast and colorectal carcinoma models was clearly visualized as early as 24 h pi [123]. The calculated absorbed dose of [^89^Zr]Zr-nimotuzumab was lower than what was earlier estimated for [^89^Zr]Zr-DFO-panitumumab [121], suggesting that [^89^Zr]Zr-nimotuzumab might be a promising alternative to panitumumab and also cetuximab [123]. A recent study also reported ^89^Zr labeling of nimotuzumab with a SpyTag/SpyCatcher, which would also enable labeling with ^225^Ac for radioimmunotherapy [124]. Currently, [^89^Zr]Zr-nimotuzumab is investigated in a phase I/II trial to determine its diagnostic quality in lung and colorectal cancer patients (NCT04235114).

As already mentioned, patient selection for HER1 targeted therapy is the major objective for development of anti-HER1 imaging probes. Perk et al. had earlier shown promising capabilities of [^89^Zr]Zr-cetuximab as a predictor for [^177^Lu]Lu- or [^90^Y]Y-cetuximab radioimmunotherapy (RIT) in A431 xenografts [125]. Despite the advances in the development of [^64^Cu]Cu- and [^89^Zr]Zr-cetuximab for imaging, preclinical and clinical data indicate that the predictive power of cetuximab imaging for therapy response remains unclear and the correlation between cetuximab uptake and HER1 expression in vivo is controversial. In 2007 Cai et al. reported the first quantitative PET of HER1 receptors in xenograft bearing mice with good correlation between the uptake of [^89^Zr]Zr-cetuximab and expression levels. This was in direct contradiction to the study later published by Aerts et al. [126,127]. Since then, several studies with radiolabeled cetuximab and panitumumab have been published supporting either side [114,116,121,128,129]. It is suggested that these under- and overestimations of HER1 expression using radiolabeled anit-HER1 antibodies could be due to different tumor models with different receptor turnover rates, vascular density, permeability, dosing and pharmacokinetics (long presence in blood and penetration ability of antibodies) [52,126,128]. One study with wild type RAS metastatic colorectal cancer (mCRC) showed an uptake of [^89^Zr]Zr-cetuximab in 6 of 10 patients, of which 4 patients had clinical benefit from cetuximab therapy [75]. In a slightly larger study with patients also suffering from RAS wild type mCRC, no correlation between the uptake and treatment benefit was observed [130]. Imaging agents that are smaller in size might provide better properties to circumvent some of these problems.

### 3.2. Antibody Fragments, scFv and sdAb for Imaging of HER1 Expression

**Figure 2:**
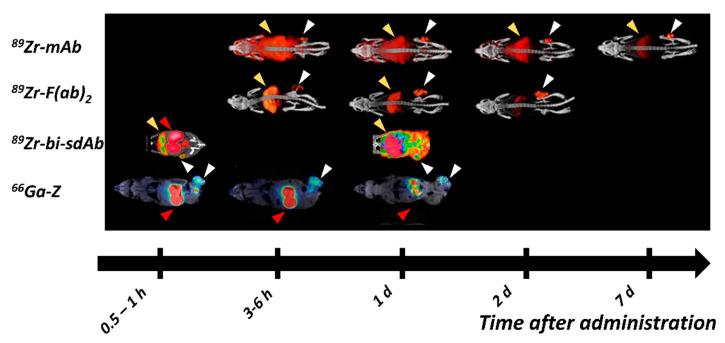
Imaging of HER1 expression (white arrows) in xenografted mice using ^89^Zr-labeled monoclonal antibody (cetuximab [87]), F(ab′)_2_-fragement (cetuximab-F(ab′)_2_ [87]), biparatopic dimer of single domain antibodies (sdAb) [135], and ^66^Ga-labeled affibody molecule (Z_2377_ [138]). Yellow arrows point to the uptake in liver and red arrows in kidneys. These images were originally published in J Nucl Med [87] © SNMMI; Sci Rep [135] and Pharmaceutics [138].

Cetuximab- and panitumumab-derived F(ab′)_2_ and Fab fragments have shown promising characteristics as imaging agents with similar affinities, but lower liver uptake and much faster clearance from blood than full length antibodies [87,131]. Good visualization at earlier time points (e.g., 24 h pi) with higher tumor-to-blood and tumor-to-liver ratios were achieved in pre-clinical studies despite lower tumor uptake [87,131]. [^111^In]In-cetuximab-F(ab′)_2_ showed good correlation between HER1 expression and uptake in HNSCC tumor models and was also able to image changes in HER1 expression induced by radiation as well as treatment with cetuximab [87,132,133]. [^64^Cu]Cu-NOTA-panitumumab-F(ab′)_2_ showed clear visualization and optimal imaging of PANC-1 xenografts 48 h pi [131]. An illustration of the differences between HER1 imaging with full-length antibodies and F(ab′)_2_ fragments can be found in Figure 2.

Panitumumab-Fab fragments labeled with ^64^Cu and ^99m^Tc-tricarbonyl have shown similar advantages in tumor-to-blood ratios over full-length antibodies, but their biodistributions are accompanied by a dramatically increased kidney uptake [131]. The tumor uptake of [^99m^Tc]Tc-Pm-Fab-His_6_ at 24 h pi was comparable with the uptake of [^64^Cu]Cu-NOTA-panitumumab-F(ab′)_2_ in PANC-1 xenografts at both 24 h and 48 h pi [131,134] and even with full-length [^111^In]-DOTA-panitumumab 72 h pi [122]. [^99m^Tc]Tc-Pm-Fab-His_6_ showed clear visualization of a panel of different HER1 expressing xenografts using SPECT. The tumor-to-blood ratio for [^99m^Tc]-Pm-Fab-His_6_ at 24 h pi (3.3 ± 0.2) was similar to the tumor-to-blood ratio for [^64^Cu]Cu-NOTA-panitumumab-F(ab′)_2_ [131].

Not many tracers, which are based on the smaller engineered antibody fragments, have been reported in the last few years. A ^99m^Tc-labeled single chain dimer (scFv)_2_, binding to domain I and II of the HER1 receptor, showed a maximum tumor uptake of 2.4 ± 0.5% IA/g (A431 xenografts), and tumor-to-blood ratios well below 0.5, both of which are lower compared with F(ab′)_2_ or Fab fragments [87,91,131,134]. Liver uptake was around 6 %ID/g at 6 h pi and increased with time, while the tumor uptake was stable. These suboptimal features translated into SPECT images, providing the highest, but still suboptimal, contrast (6 h pi), requiring further optimization of the probe.

Technetium labeling has also been used to label HER1-targeting sdAb for SPECT, showing much more promising characteristics than (scFv)_2_ with quick blood clearance and high tumor accumulation [135,136]. [^ 99m ^ Tc]Tc-D10 was able to detect HER1 expressing lesions already 45 min pi ( Figure 2) [135]. Another promising candidate might be Df-Bz-NCS-7D12 labeled with ^68^Ga and ^89^Zr, which provides a higher tumor uptake than [^ 99m ^ Tc]Tc-D10 and good visualization on PET 1h pi [137]. Tumor-to-blood ratios for both constructs were comparable to [^64^Cu]Cu-NOTA-panitumumab-F(ab′)_2_ and [^111^In]In-cetuximab-F(ab′)_2_.

### 3.3. Affibody Molecules for Imaging of HER1 Expression

Affibody molecules are currently the only class of ESPs investigated for molecular imaging of HER1 expression. In total, three different HER1-targeting affibody molecules have been characterized for imaging purposes in preclinical studies: Z_EGFR:03115,_ Z_EGFR:1907,_ Z_EGFR:2377_ [139,140,141]. Both Z_EGFR:03115_ and Z_EGFR:1907_ have lower affinities to HER1 than Z_EGFR:2377_, which has been the most studied variant for imaging. Comparison of anti-HER1 affibody molecules with Fab- or F(ab′)_2_-fragments is difficult, due to the lack of information on cross-reactivity to murine ErbB1. As mentioned earlier, the choice of a preclinical model and dosing needs to be considered when comparing different studies.

The majority of affibody-based tracers for HER1-imaging have focused on PET, but none of them have made the transition into clinical studies, yet. Compared with [^89^Zr]Zr-DFO-cetuximab 48 h pi, the affibody molecule [^89^Zr]Zr-DFO-Z_EGFR:2377_ showed quicker localization in tumors [140]. The uptake of [^89^Zr]Zr-DFO-Z_EGFR:2377_ 3 h pi (4.3 ± 0.6 % ID/g) exceeded the uptake of the mAb at the optimal imaging time, 48 h pi. While the tumor uptake of [^89^Zr]Zr-DFO-Z_EGFR:2377_ decreased from 3 to 24 h pi, the uptake at 24 h was still similar to the tumor uptake of [^89^Zr]Zr-DFO-cetuximab 48 h pi. The correlation between tracer uptake and HER1 expression (determined by IHC) was demonstrated using [^89^Zr]Zr-DFO-Z_EGFR:03115_, a different ^89^Zr-labeled HER1-targeting affibody clone [139].

Blood clearance of Z_EGFR:2377_ is slower than the clearance of affibody molecules binding to other molecular targets [120,140,142]. This, most likely, could be attributed to the substantial uptake of HER1-binding affibody molecules in the liver due to the natural receptors’ expression and slow internalization of Z_EGFR:2377_ [120]. Z_EGFR:2377_ is cross-reactive to murine ErbB1, which is a definite advantage as this allows for more accurate estimation of accumulation in non-tumor tissues. Clearance of radiolabeled Z_EGFR:2377_ from blood shifts the equilibrium for hepatocyte-bound tracers to dissociation, causing the release of bound [^89^Zr]Zr-DFO-Z_EGFR:2377_ back into the blood stream. Regardless, [^89^Zr]Zr-DFO-Z_EGFR:2377_ provided higher tumor-to-tissue contrast at both 3 h and 24 h pi than [^89^Zr]Zr-DFO-cetuximab at 48 h pi [140]. Figure 2 illustrates the imaging contrast of HER1-targeting affibody molecules at different time points, in comparison with antibodies, antibody fragments and sdAb.

[^68^Ga]Ga-DFO-Z_EGFR:2377_, 3 h pi, provided an improved tumor uptake and PET-contrast compared with [^89^Zr]Zr-DFO-Z_EGFR:2377_ in the same preclinical model (A431 xenografts) [140,142]. Imaging even earlier, e.g., 1 h pi, using [methyl-^11^C]-Z_EGFR:2377_-ST-CH_3_, proved to be disadvantageous as the uptake in xenografts was still increasing [143]. Fluorine-18 was also explored as a PET-radionuclide with a shorter half-life [141,144]. [^18^F]AlF-NOTA-Z_EGFR:03115_, 1 h pi, was able to image the downregulation of HER1 in response to cetuximab treatment in HN5 xenografts (with high HER1 expression) [139]. Nevertheless, the ^18^F-labeled affibody molecules still provided inferior contrast to ^68^Ga-, ^89^Zr- and radiocobalt-labeled affibody molecules [140,142]. Besides the excellent properties of [^68^Ga]Ga-DFO-Z_EGFR:2377,_ the observed increase in imaging contrast for [^89^Zr]Zr-DFO-Z_EGFR:2377,_ with time, indicated a potential for the use of longer lived nuclides. Z_EGFR:1907_ has been labeled with ^64^Cu, showing better PET-imaging properties than [^64^Cu]Cu-DOTA-cetuximab [127]. However, the transchelated ^64^Cu caused substantial hepatic uptake, limiting the feasibility of [^64^Cu]Cu-DOTA-Z_EGFR:1907_ for imaging [144,145]. Thus far, radiocobalt-labeled DOTA-Z_EGFR:2377_ for PET imaging provided the best contrast among all studied anti-HER1 affibody variants in mice with A431 xenografts, with an impressive tumor-to-liver ratio of 3.1 ± 0.5 at 3 h pi [105,120,139,140,141,142,145,146,147].

An advantage of affibody-based tracers is the possibility to modify the scaffold, its charge and charge distribution. This can influence their biodistribution and, in particular, affect the hepatic uptake as was shown with a panel of ^64^Cu-labeled variants of Z_EGFR1907_ [148]. This was also illustrated by Oroujeni et al., who demonstrated the liver uptake of [^99m^Tc]Tc-Z_EGFR:2377_ as reversely proportional to the number of glutamates in the peptide based chelators [146].

Z_EGFR:2377_ and Z_EGFR:1907_ were labeled with indium-111 and technetium-99m for SPECT imaging. The distribution of [^111^In]In-Bz-DTPA-Z_EGFR:1907_, [^111^In]In-DOTA-Z_EGFR:2377_, and [^99m^Tc]Tc-EEEC-Z_EGFR:2377_ in A431 was comparable, with generally improved tumor/tissue contrast 24 h pi. The low-cost and availability of ^99m^Tc generators make them very attractive, and [^99m^Tc]Tc-EEEC-Z_EGFR:2377_ is a promising alternative for SPECT-imaging. 

Another scaffold protein, [^64^Cu]Cu-Fn_EI3.4.3′_, which is based on a 10 kDa fibronectin binding domain, provided good tumor visualization using PET and superior tumor-to-blood contrast to the ^64^Cu-labeled affibody just 1 h pi [149,150] and might be another possible alternative for imaging of HER1 expression. However, this tracer has very low affinity to murine ErbB1 (2.5 µM) [149,150]. Thus, the model does not reflect the important interaction of the tracer with hepatocytes.

### 3.4. Small Peptides

There are a number of agents smaller than ESPs that should be mentioned. The GE11 peptide was first reported in 2005. GE11 binds HER1 on the same epitope as EGF without triggering mitogenic activity [151]. Even though it appears that GE11, to some extent, also binds to HER2 [152], it has been evaluated for its HER1 imaging ability [153,154,155]. It has been labeled with ^99m^Tc, ^111^In, ^18^F, and ^64^Cu. [^64^Cu]Cu-NOTA-linker-β-Ala-GE11 for PET-imaging failed to show sufficient uptake and contrast in FaDu xenografts, which was presumed to be due to peptide aggregation [155]. The most recent data for [^18^F]FP-Lys-GE11 [154] and ^99m^Tc-peptide-GE11 [153] in U87, PC3, A431, and A549 xenograft models suggest the most suitable imaging time point to be 2 h pi when both peptides have reached maximum tumor uptake. However, high uptake in the abdomen, particularly in the liver, intestine, and stomach, exceed or match uptake in tumors and limits the imaging contrast.

It should briefly be noted that molecular imaging of HER1 point mutations (e.g., exon 19 E746-A750 deletion or exon 21 L858R point mutation) has also been reported, mostly aimed towards the selection of patients for TKI therapy. Tracers are often TKI-derived and have shown potential for patient selection in preclinical and clinical studies [156,157,158]. Activating EGFR mutant kinase were detected using ^18^F-MPG in patients with non-small cell lung cancer (NSCLC) and it was demonstrated that patients with a high uptake of ^18^F-MPG (SUV > 2.23) had a greater response to EGFR-TKIs (>80% versus 6%) and twice longer median progression-free survival [156]. The complexity in tumor biology (presence of tumor heterogeneity, cancer stem cells, hypoxia and many other factors) provides a precondition for the development of a wide panel of imaging agents that could add to diagnostic understanding and improve precision treatment [159,160].

### 3.5. HER1 Concluding Remarks

In summary, many tracers of different formats are currently being explored for imaging of HER1 expression (Table 1). ^89^Zr-and ^64^Cu-labeled mAbs that have been available for a rather long time are currently in clinical trials, but have yet to be approved for routine use. High hepatic tracer accumulation limits the detectability of lesions in the liver and the abdomen. Data concerning the correlation of uptake and receptor’s expression and, consequently, PET-based discrimination between lesions with high and low expressions for patient selection are still inconclusive. Smaller imaging probes, particularly F(ab′)_2_ fragments, sdAb and affibody molecules, show great promise in preclinical studies, providing an improved tumor-to-tissue contrast at earlier time points, and should be pursued in translational studies.

## 4. HER2 Imaging

HER2 is probably the most explored target for molecular imaging among the EGFR-family receptors. Targeted treatment of HER2-positive breast and gastric cancers substantially improves the survival of patients [163,164]. However, only in situ hybridization (ISH) positive patients or patients with HER2 expression of 3+ according to IHC are considered to be clinically HER2-positive and eligible for HER2 targeted treatment. The number of HER2 receptors in tumors is around ~2.3 × 10^6^ receptors/cell in these patients [165], which is substantially higher compared with what are considered high levels of HER1 and HER3 overexpression [30,166]. Moreover, patients with a HER2 IHC status of 1+ or 2+, who are considered clinically HER2-negative, still express a considerable amount of HER2 (10^5^ and 5 × 10^5^ receptors per cell, respectively) [165]. Thus, the major challenge for the development of HER2-targeting radiotracers would be to differentiate reliably between the different HER2 expression levels in malignant lesions if molecular imaging of HER2 expression is to be applied for a selection of patients for treatment. The very low expression of HER2 in healthy tissue, compared with HER1 and HER3, is an advantage for good imaging contrast. Still, it is important to keep in mind that a non-negligible amount of HER2 is expressed by hepatocytes [167]. Clinical data suggest that it is necessary to co-inject a sufficient mass of a non-labeled probe with the tracer to saturate HER2 receptors in the liver and enable a high imaging contrast [78,168,169].

### 4.1. Monoclonal Antibodies

The existing therapeutic HER2-targeting mAbs trastuzumab and pertuzumab were the first choice for HER2-targeting molecules to be radiolabeled for PET and SPECT imaging [84,170,171,172]. Perik et al., in 2006, reported on HER2 scintigraphy with [^111^In]In-MxDTPA-trastuzumab in metastatic breast cancer (mBC) patients [171]. While new tumor lesions were detected in 13/15 patients, the overall detection rate was only 45% for known lesions identified by IHC. Progress in the development of ^111^In-labeled HER2-targeting antibodies for SPECT has been limited despite further research efforts [173,174,175,176] due to the suboptimal contrast and sensitivity. The conjugate [^111^In]In-CHX-A”-DTPA-trastuzumab was recently evaluated in a Phase 0 study showing safety, but no data on tumor targeting are yet available [177].

**Figure 3:**
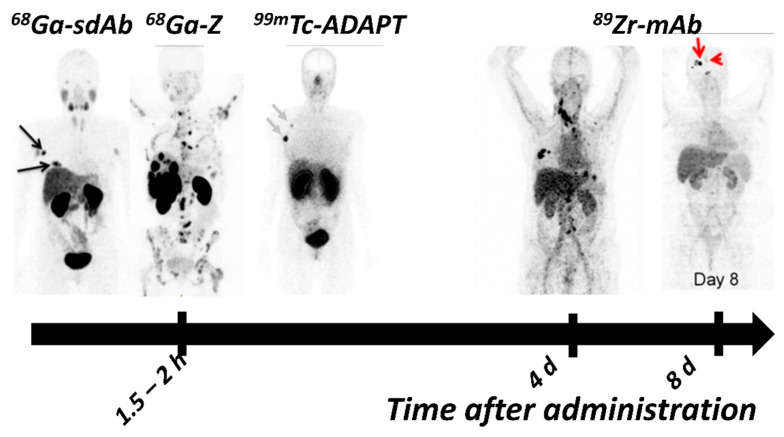
Imaging of HER2 expression in patients using ^68^Ga-sdAb [71,90] and affibody ([^68^Ga]Ga-ABY-025 [78], abbreviated ^68^Ga-Z) and ABD Derived Affinity Proteins (ADAPT) labeled with ^99m^Tc [169], and ^89^Zr-labeled mAbs trastuzumab (left [57]) and pertuzumab (right [182,191]. These images were originally published in JNM [57,169,182,204] © SNMMI, Theranostics [78].

PET-imaging with ^89^Zr- and ^64^Cu-labeled variants of both mAbs are mostly dominating the preclinical and clinical development of HER2 targeting antibody-based imaging agents. The first clinical proof-of-concept study of HER2-PET-imaging with ^89^Zr-labeled trastuzumab, published by Dijkers et al. in 2010, included 14 patients with HER2-positive mBC [74]. Researchers were able to identify HER2-positive lesions, and the best possible image contrast was achieved 4-5 d pi. Since then, many other clinical studies have demonstrated the feasibility of [^89^Zr]Zr-trastuzumab and [^89^Zr]Zr-pertuzumab imaging [178,179,180,181,182]. Additionally, one study even reported the benefit of [^89^Zr]Zr-trastuzumab PET for clinical decision-making [57]. Examples of clinical images with ^89^Zr-labeled mAb for HER2 PET can be seen in Figure 3.

The detection of HER2-positive metastases in case of HER2-negative primary cancer using [^89^Zr]Zr-trastuzumab was addressed by Ulaner et al. in two related, consecutive clinical studies including a total of 20 patients [181,183]. Unfortunately, HER2-positive metastases could only be correctly identified in 15% of patients, while in 30% of patients, suspicious foci from PET-imaging proved to be false-positives. Similar results were found in a matching study with [^89^Zr]Zr-DFO-pertuzumab [184]. The reasons are still unclear but could be related to heterogeneity, pathological sampling errors, non-specific accumulation of free ^89^Zr (particularly problematic in bone metastases), or the fast internalization of trastuzumab and pertuzumab in combination with the late time point imaging necessary for blood clearance of antibody-based tracers. While imaging of breast cancer patients still dominates HER2 imaging, O’Donoghue et al. reported the first use of [^89^Zr]Zr-trastuzumab in 10 esophagogastric cancer patients, where up to 80% of the known HER2-positive lesions were visualized [74,179,180].

Besides [^89^Zr]Zr-trastuzumab and [^89^Zr]Zr-pertuzumab, their ^64^Cu-labeled analogs have successfully been applied for imaging of HER2 expression. A side-by-side preclinical comparison of [^89^Zr]Zr-DFO- and [^64^Cu]Cu-DOTA-trastuzumab indicated a higher tumor uptake of [^89^Zr]Zr-DFO-trastuzumab [172]. However, its higher background uptake led to comparable tumor-to-tissue ratios. The first feasibility study of [^64^Cu]Cu-trastuzumab in humans included six patients with BC, and was reported by Tamura et al. in 2013 [185]; primary tumors and brain metastasis, down to a size of 2x2 cm, could be visualized. The optimal imaging time point with [^64^Cu]Cu-DOTA-trastuzumab is considered to be 48 h pi. Although, another study including eight patients with mBC reported that most lesions could be visualized already 24 h pi using [^64^Cu]Cu-trastuzumab, and the uptake correlated with the HER2 status [186,187]. The earlier time point means a higher tracer concentration in the background signal from blood, making it more difficult to detect possible lesions close to or inside well-perfused organs, such as the liver [185,186]. The detection rate increased from 77% to 89% on the second day, which was mainly due to the visualization of additional lymph nodes and hepatic lesions (this probably indicates that radionuclides with longer half-lives are required for immunoPET) [186]. Interestingly, the detection sensitivity for [^64^Cu]Cu-trastuzumab 48 h pi was only marginally lower than for FDG (93%) [186]. An increase in the injected protein dose by 45 mg reduced the activity retention in the liver appreciably (up to 75%), with no major effect on tumor uptake [186]. Co-injection of unlabeled mAb is generally required to improve the image quality and is settled in the range of 45–50 mg for patients without HER2-targeted treatment and 10 mg in treated patients [74,179,183]. However, when another group attempted imaging with [^64^Cu]-DOTA-trastuzumab in 11 patients after a therapeutic dose of 50 mg trastuzumab, only a small fraction of the lesions were detectable [188].

Aside from the radiometal labels, [^124^I]I-trastuzumab was used for PET-imaging in a small clinical study involving six patients after [^124^I]I-trastuzumab had demonstrated a higher imaging contrast than [^64^Cu]Cu-NOTA-trastuzumab in a preclinical model due to a lower nonspecific uptake [170,189]. In clinics, [^124^I]I-trastuzumab could visualize HER2 expression in two of the two HER2-positive patients. However, imaging needs to be done at earlier time points than with the ^89^Zr-labeled trastuzumab, e.g., 24 h and 48 h. The non-residualizing properties of the iodine-label resulted in an elevated background and loss of the HER2-specific signal from tumors at later time points [170]. Therefore, the use of radioiodine labels for rapidly internalizing HER2-targeting molecules such as trastuzumab and pertuzumab [190] might be suboptimal [191,192]. Using more slowly internalizing targeting molecules, such as affibody molecules, could improve imaging with radioiodine in comparison with iodinated trastuzumab, because the intracellular degradation of the molecules and release of free iodine into the blood should be slower in this case [191].

Imaging of the response to HER2-targeted trastuzumab and pertuzumab therapy is a question of high clinical interest. Gebhart et al. investigated the correlation between the uptake of [^89^Zr]Zr-trastuzumab and treatment outcomes of trastuzumab-emtansine (T-DM1) therapy in 60 patients with mBC as part of the ZEPHIR trial [56]. It was shown that patients with [^89^Zr]Zr-trastuzumab PET-positive lesions and a reduction in metabolic activity after the first treatment cycle had a better response to T-DM1 treatment in terms of tumor shrinkage and progression-free survival. Interestingly, a preclinical study with [^89^Zr]Zr-pertuzumab showed no changes in SUV values for neither [^18^F]FDG nor [^89^Zr]Zr-pertuzumab after T-DM1 therapy of mice with BT474 xenografts, despite treatment-induced xenograft shrinkage. [^89^Zr]Zr-pertuzumab was, however, able to visualize the reduction in tumor size in mice [193].

Dosimetry studies for [^89^Zr]Zr-trastuzumab and [^89^Zr]Zr-pertuzumab revealed similar dosimetry profiles with liver being the critical organ. Reported effective doses for [^89^Zr]Zr-trastuzumab and [^89^Zr]Zr-pertuzumab are comparable (0.48 mSv/MBq vs 0.54 mSv/MBq), but higher than for their ^64^Cu labeled counterparts (also the liver being the organ with the highest absorbed dose) [179,182,185,194]. In addition to the elevated liver doses, the detection of liver metastases was rather difficult and somewhat unreliable, due to the involvement of normal liver tissue in the mAb catabolism [74,185,195]. For example, in the first proof-of-concept study with [^89^Zr]Zr-trastuzumab, liver lesions were missed in 3 out of 7 patients [74]. In another study, the SUVmax values for HER2-positive and negative lesion were significantly different (*p* < 0.05) only after the exclusion of hepatic lesions [195].

The success of radiolabeled trastuzumab and pertuzumab in clinical trials has not stopped the preclinical development of improved antibody-based probes for HER2 imaging. For example, modifications of the DFO chelator, used for the labeling of trastuzumab and pertuzumab with ^89^Zr, resulted in the DFO-derivative DFO* [196]. DFO* was conjugated to trastuzumab and labeled with ^89^Zr to evaluate its potential [197]. [^89^Zr]-DFO*-trastuzumab showed improved stability of the radiolabel, which was particularly indicated by the significantly lower uptake in the liver and bone compared with the uptake of its DFO conjugated counterpart in these organs. No effects on the overall distribution, blood kinetics and tumor uptake were observed. Most impressively, the uptake in bone of [^89^Zr]-DFO*-trastuzumab decreased from 24 h to 144 h pi by approximately two-fold. Similar results were found by Cho et al. [198] studying the delivery of T-DM1 to tumors, suggesting that [^89^Zr]Zr-DFO*-T-DM1 would be more useful than [^89^Zr]Zr-DFO-T-DM1. Site-specific conjugation of DFO (to ensure a uniform product with a minimal loss of immunoreactivity) and subsequent labeling with ^89^Zr resulted in a higher uptake in tumors, but also a higher concentration in blood compared with randomly labeled trastuzumab, and did not show any advantages [199].

A study aiming to improve the stability of [^64^Cu]Cu-DOTA-trastuzumab and reduce the hepatic accumulation of free ^64^Cu investigated the use of a NOTA chelator instead of DOTA [189,200]. The [^64^Cu]Cu-NOTA label indicated good stability, and in vivo hepatic uptake was lower than what was previously reported for [^64^Cu]Cu-DOTA-trastuzumab [172,200,201]. Guo et al. additionally studied [^64^Cu]Cu-NOTA-trastuzumab in two patients, where liver metastases >1 cm were visualized. Another trastuzumab-conjugate with a novel chelator for copper, [^64^Cu]Cu(Sar), showed promising tumor targeting and imaging in preclinical models [202]. On this note, Tolmachev et al. [203] found NOTA and its derivative NODAGA to be suboptimal chelates for the labeling of affibody molecules with copper. However, the suboptimal biodistribution could most likely be caused by the very high reabsorption of affibody molecules in kidneys and re-distribution of renal radiometabolites. Larger studies, and possibly a proper side-by-side comparison of the conjugates, would be needed to obtain a reliable conclusion as to which conjugate is better suited for the imaging of HER2 expression.

### 4.2. Antibody Fragments for Imaging of HER2 Expression

**Figure 4:**
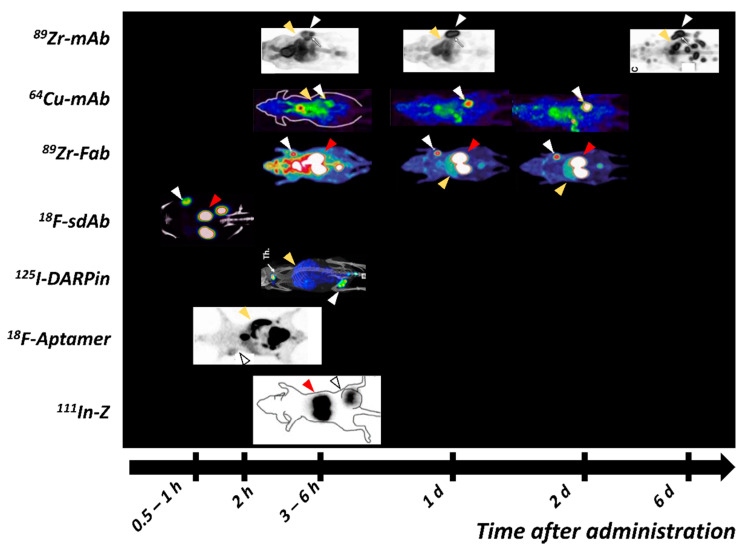
Imaging of HER2 expression (white arrows) in xenografted mice using monoclonal antibodies ([^89^Zr]Zr-trastuzumab [84] and [^64^Cu]Cu-pertuzumab [194]), [^89^Zr]Zr-Fab (PASylated fragments of trastuzumab [207], ^18^F-labeled sdAb [217], ^125^I-labeled DARPin (G3 [224], ^18^F-labeled aptamer [225], and ^111^In-labeled affibody molecule (ABY-025 [226], abbreviated ^111^In-Z). Yellow arrows point to uptake in the liver and red arrows in the kidneys. These images were originally published in JNM [84,207,217,226] © SNMMI, Int J Mol Sci [224], and PLoS One [225].

Studies of proteolytically produced fragments of trastuzumab and pertuzumab showed an overall lower tumor uptake compared with their full-length counterparts due to faster elimination from the blood stream and lower bioavailability [168,205,206]. However, the use of antibody fragments enabled good imaging contrast already at 24 h pi. Lam et al. showed that [^64^Cu]Cu-NOTA-pertuzumab F(ab′)_2_ can detect trastuzumab-induced changes in HER2 expression in a preclinical model [205]. Mendler et al. studied a PASylated trastuzumab Fab-fragment labeled with ^89^Zr and ^124^I. The authors demonstrated that both tracers showed comparable contrast and good visualization of xenografts 24 h pi despite the lower tumor uptake of the ^124^I-labeled variant (Figure 4) [207]. Recently, [^89^Zr]Zr-Df-Fab-PAS200 was tested in the first clinical PET study. The lesions were detectable at 24 h pi, with one of them supposedly being the previously undetected primary tumor [208]. The uptake in the kidney and hepatobiliary system dominated the images.

Even earlier imaging with [^68^Ga]Ga-DOTA-F(ab′)_2_-trastuzumab could detect changes in HER2 expression in BT474 xenografts, with the imaging signal intensity being proportional to HER2 expression [206]. However, in the clinical pilot study using [^68^Ga]Ga-DOTA-F(ab′)_2_-trastuzumab, HER2-positive lesions were only detected in half of the patients with a known HER2-positive disease [209]. The authors speculated that suboptimal dosing could affect the contrast, as all patients received the same dose independent of their trastuzumab treatment histories.

### 4.3. scFv, Diabody, Minibody, sdAb for Imaging of HER2 Expression

Initially, anti-HER2 scFv demonstrated low tumor accumulation (0.7–1%ID/g), which could potentially be explained by the choice of a non-residualizing iodine label [210,211,212]. In addition, mathematical modeling suggests that the globular proteins with a molecular weight of about 25 kDa provide the lowest tumor uptake [212]. Good tumor-to-blood ratios (in the range of 9 to 15) were reported despite low tumor accumulation. Probes based on dimeric forms of scFv, such as (scFv)_2_ and diabodies, showed a 2–2.7 times higher uptake in xenografts, and even higher tumor-to-blood, tumor-to-liver and tumor-to-bone ratios [213,214]. Labeling of a scFv with [^68^Ga]Ga-Df-p-SCN [215] also significantly improved tumor uptake compared with the tumor uptake of ^125^I-labeled scFv, but this tracer had lower tumor-to-tissue ratios. ^111^In-labeled diabodies and minibodies showed the best characteristics for HER2 imaging among scFv and scFc-derivatives [214,216]. However, their capabilities appear subpar compared with the many other types of available engineered antibody fragments and engineered scaffold proteins, so their future for HER2 imaging is questionable.

Several HER2-targeting sdAb have been radiolabeled and evaluated as imaging probes [217,218]. Among these, 2Rs15d sdAb (later also called Anti-HER2-VHH1 [219]) is the most promising variant, providing uptake in the range of 4–19 %ID/g in SKOV-3 and BT474 xenografts 1–3 h pi [218,220,221] (Figure 4). The observed high uptake at early time points has enabled the labeling of 2Rs15d with short lived labels, such as ^18^F [221,222], ^68^Ga [218], ^99m^Tc [218] and ^131^I [220], providing tumor-to-blood ratios higher than radiolabeled trastuzumab and pertuzumab several days after injection. The uptake of sdAb in the kidney is substantially elevated, particularly in the case of residualizing labels, but it appears that it could be reduced by the removal of a hexahistidine-tag in the sdAb sequence [218]. Two variants of 2Rs15d have been reported in clinical trials: [^68^Ga]Ga-HER2-Nanobody [223] and [^131^I]I-GMIB-Anti-HER2-VHH1 [219]. The Phase I study of [^68^Ga]Ga-HER2-Nanobody in 10 BC patients demonstrated a well-tolerated administration and the ability of the sdAb probe for imaging of HER2 expression in primary tumors and metastases 6–90 min pi (Figure 3). Due to tracer clearance from normal tissues, 90 min pi was suggested as the more optimal time point. High uptake was observed in the kidney due to renal tracer elimination [223]. [^131^I]I-GMIB-Anti-HER2-VHH1 is envisioned as a theranostic agent. The recent clinical study by D’Huyvetter et al. evaluated safety, distribution, dosimetry and tumor imaging, showing uptake in patients with lesions >3 cm; the visualization of smaller lesions was difficult, supposedly due to PVE [219]. Both clinical studies included only a small number of patients and warrant further clinical investigations, which are currently ongoing (NCT03924466, NCT03331601).

### 4.4. ESPs for Imaging of HER2 Expression

Affibody molecules, ADAPTs, and DARPins selectively targeting HER2 have been developed, and have shown excellent characteristics for the imaging of HER2-positive malignant diseases in preclinical and clinical studies [78,94,95,169,224]. The affibody molecule Z_HER2:342_ (and its derivatives), ADAPT6, and the G3 DARPin are the most promising and the most studied variants for HER2 targeting within their respective classes of ESPs. Unlike trastuzumab and pertuzumab, the studied HER2-targeting ESPs are slowly internalized by cancer cells [86,94,227]. The slow internalization increases the need for a strong interaction between HER2 and the targeting molecule with high affinity and particularly slow off-rates, to ensure good agent retention in tumors. The highest affinities (the lowest equilibrium dissociation constants) reported for anti-HER2 affibody molecules, ADAPT6 and G3 DARPin were 29 pM [228], 2.5 nM [94], and 90 pM [229], respectively. Noticeably, the affinity of ADAPT6 is in the low nanomolar range, but studies with affibody molecules have indicated that such an affinity is sufficient for efficient tumor uptake 4 h pi for targets with high expression [103]. Interestingly, dimerization of ESPs has shown to increase the affinity, but not the total tumor uptake of anti-HER2 affibody molecules and ADAPTs [230,231], as was the case for scFv and (scFv)_2_ discussed above.

The affibody molecule Z_HER2:342_ is the most investigated clone in preclinical studies among HER2-targeting ESPs. Its synthetic DOTA-conjugated variant ABY002 was labeled with ^111^In and ^68^Ga and studied in a clinical proof-of-principle study including three patients with mBC [232]. This initial study demonstrated the safety and ability of [^111^In]In- and [^68^Ga]Ga-ABY002 to detect HER2-positive metastases as early as 2–3 h pi. Nine out of eleven [^18^F]FDG-positive metastases were detected: a higher rate than in the first studies with [^111^In]In-DTPA-trastuzumab [171]. As expected, a high uptake was observed in kidneys, but, somewhat unexpectedly, also in the liver, which was not indicated by preclinical studies. Z_HER2:342_ was reengineered with an exchange of 11 surface-exposed amino acids (none of them involved in binding) to optimize the composition for peptide synthesis, storage stability, higher melting point, and surface hydrophilicity, resulting in the new scaffold [226]. The second generation anti-HER2 affibody, ABY025 (DOTA-Z_HER2:2891_) was labeled with ^111^In and SPECT-imaging was performed in seven patients with mBC [233]. Some clinically HER2-negative lesions also showed an elevated uptake. The uptake in the HER2-positive lesions increased with time and the ratio between the uptakes at 24 h and 4 h pi was proposed as a diagnostic tool to separate clinically HER2-positive from clinically HER2-negative lesions. In addition, some HER2-positive metastases, that were previously not identified by [^18^F]FDG-PET could clearly be visualized on the [^111^In]In-DOTA-ABY025 SPECT. In another study, [^68^Ga]Ga-ABY025 demonstrated a correlation between the SUV in lesions with a confirmed HER2 IHC score, excellent repeatability of PET scans, and the influence of the peptide dose on image quality [78]. Increasing the injected dose by the addition of unlabeled ABY025 lowered the activity uptake in the liver and led to an improved visualization of hepatic metastases. The treatment plan was changed after results of the [^68^Ga]Ga-ABY-025 scan in 3 of 16 patients. Kidneys, the excretory organ for most ESPs, remained the organ with the highest uptake, followed by the liver. However, a metastasis in an adrenal gland was detected, despite the close location to the kidneys. The overall radiation dose was estimated to be 0.028 mSv/MBq; that is 20-fold lower than for ^89^Zr-labeled mAbs [179,182]. A multicenter Phase II/III study of [^68^Ga]Ga-ABY025 is currently underway (NCT03655353).

Recently, extensive studies on Z_HER2:342_ modified with an N-terminal Cys- Gly–Gly–Gly–Arg–Asp–Asn linker, entitled MZHER2:342, were reported [234]. Authors aimed to increase the hydrophilicity and reduce the background in the abdomen. MZHER2:342 was labeled with [^18^F]AlF-NOTA, ^68^Ga, and ^89^Zr, enabling clear visualization of SKOV3 and BT474 xenografts as early as 30 min pi in preclinical studies [234,235,236]. Evaluation of [^68^Ga]Ga-NOTA-MAL-Cys-MZHER2:342 in two mBC patients showed that HER2-positive lesions could be detected and suggested a lower SUV uptake in the liver tissue compared with [^68^Ga]Ga-ABY025 and ABY002 [78,232,233]. In a clinical trial including 34 patients with gastric cancer, the optimal contrast for imaging with [^68^Ga]Ga-NOTA-MAL-Cys-MZHER2:342 was achieved 2 h pi. The authors reported 100% specificity and 55% sensitivity at an SUV cut-off at 6.6 [237].

Affibody molecules are no longer the only HER2 targeting ESPs under clinical investigation. In 2020, Bragina et al. reported the first clinical study for SPECT imaging of HER2 expression in BC patients (29 patients) with [^99m^Tc]Tc-ADAPT6 [169]. The administration was well tolerated. Effective doses of 0.009 ± 0.002 (500 µg) and 0.010 ± 0.003 mSv/MBq (1000 µg) were reported. The optimal injected protein dose was 500 µg. The use of this dose enabled differentiation between clinically HER2-positive and HER2-negative lesions already 2 h after injection. In one patient, [^99m^Tc]Tc-ADAPT6 was able to visualize bone metastases in the rib cage and spine, which were not detected by the CT or [^99m^Tc]Tc-pyrophosphate bone scan. These lesions were confirmed later using MRI.

DARPin is another ESP evaluated for the theranostic targeting of HER2 [238]. Two variants of DARPin, 9_29, consisting of five ankyrin repeats, and G3, consisting of four ankyrin repeats, were directly compared in vitro and in vivo [86]. Binders were labeled with radioiodine and technetium-99m. The shorter variant G3 demonstrated a 3-fold higher tumor uptake and lower uptake in healthy tissue, despite the affinity being in the same range. The influence of the position and composition of histidine-containing tag on the targeting and imaging properties of anti-HER2 DARPin G3 has been investigated in follow up studies. The scaffold was labeled with technetium and radioiodine [224,239,240]. It was demonstrated in a preclinical setting that [^99m^Tc]Tc-(HE)_3_-G3 is an optimal molecular format for SPECT-imaging. Recently, [^99m^Tc]Tc-(HE)_3_-G3 has entered the clinical stage, and a trial to assess safety, distribution, and dosimetry in patients with primary breast cancer is currently ongoing (NCT04277338).

### 4.5. Aptamers and Peptides

HER2 has no known natural high affinity ligands that could be used as imaging probes. In the last few years, there has been a small surge in studies reporting radiolabeled short peptides (6–15 amino acids) and aptamers targeting HER2 for imaging. Maximum uptake is generally achieved within 15–30 min pi, but it is rather low (<2%ID/g). Most likely a poor affinity and quick activity washout from tumors result in low tumor-to-tissue contrast compared with antibodies, antibody fragments and ESPs [225,241,242,243,244,245,246,247,248]. Nonetheless, [^99m^Tc]Tc-HYNIC-H10F peptide was administered to two BC patients (1 HER2-positive, one HER2-negative) and could identify HER2 expression in breast tissue of the HER2-posive patient [247]. Otherwise noteworthy, in a gastric cancer model (N87 xenografts) [^111^In]In-DTPA-AHNP-PEG has shown a higher tumor uptake and tumor-to-tissue ratios than other reported HER2 targeting peptides [242]. 

### 4.6. HER2 Concluding Remarks

Table 2 summarizes clinically and preclinically studied PET and SPECT imaging tracers discussed above. Taken together, radiolabeled trastuzumab and pertuzumab have led the development of radiotracers for the imaging of HER2 expression and have been studied extensively in clinics. However, the recent success of smaller probes could be a potential game changer for HER2 imaging. sdAb and three types of ESP-based tracers (affibody, ADAPT, DARPin) are in clinical trials showing extremely promising results and ability to distinguish between HER2 expression levels. Despite increased efforts towards the development of radiolabeled peptides for imaging of HER2, they currently do not appear to have the ability to challenge the position of antibody- or ESP-based tracers as the tracers of choice for HER2 imaging.

## 5. HER3 Imaging

HER3 is probably the most challenging imaging target within the EGFR family because of the relatively low and dynamic overexpression in cancer cells and substantial natural expression in healthy tissue. Since the development of HER3-targeting therapeutic agents is still in an early phase compared with the development of HER1 and HER2, it is not surprising that this also applies for the development of radiotracers for the detection of HER3 expression. However, lessons learned from HER1 and HER2 tracer development may influence and accelerate the development of HER3-targeting imaging agents. Even in its early stages, HER3 tracer development seems to be much more uniformly spread across different classes of targeting molecules. This may be due to prior experience from HER1 and HER2 targeting tracers, but also because the requirements for successful tracers are more demanding for such a challenging target as HER3. The level of HER3 expression in normal tissues is non-negligible and comparable to that of HER1 while the level of HER3 expression on cancer cells is two orders of magnitude lower, typically below 50,000 receptors per cell [30]. HER3 presented in normal tissues (organs of the gastrointestinal tract, salivary glands, lung, and especially liver) might efficiently sequester HER3 targeting imaging probes from blood circulation, preventing the probe from reaching the tumor. An imaging agent that was not internalized or catabolites could leak back into blood, which should decrease imaging contrast. The low HER3 expression in tumors strongly restricts the optimal injected dose of the imaging agent (the approach that was successfully used for HER1 and HER2 imaging). Therefore, it is important to develop a probe with a high affinity to HER3 and low off-target uptake in normal organs. Cross-reactivity of the probe to murine ErbB3 is an important aspect to be considered during interpretation of pre-clinical data.

### 5.1. Monoclonal Antibodies and Antibody Fragments for Imaging of HER3 Expression

**Figure 5:**
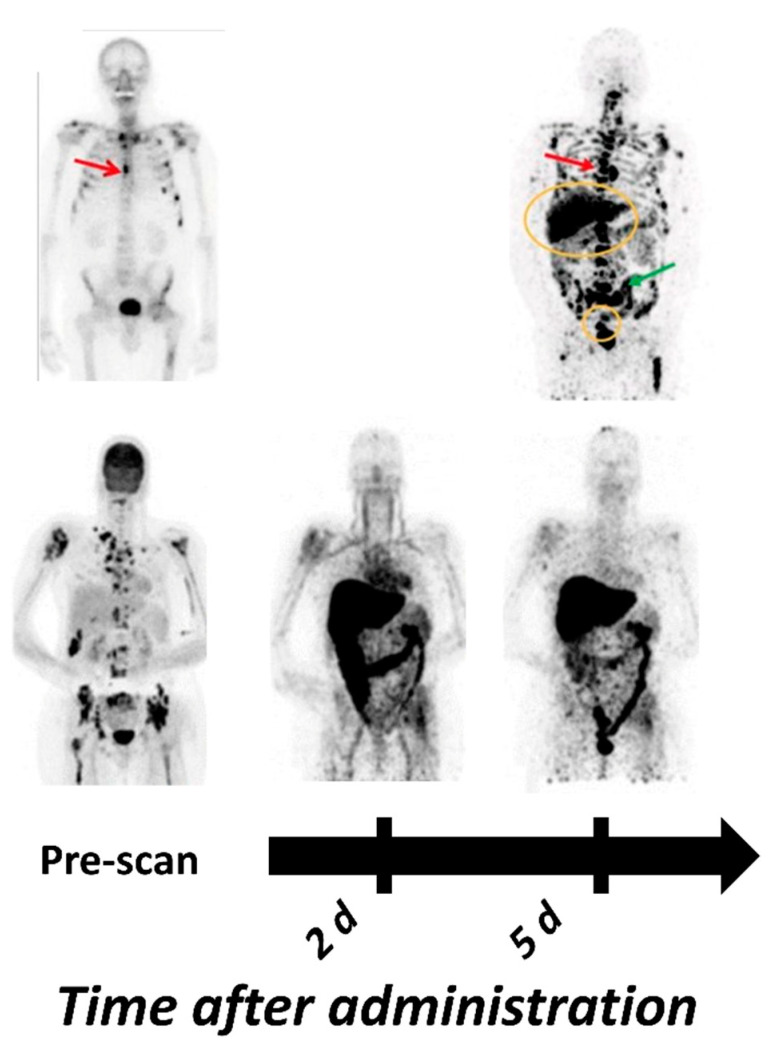
Imaging of HER3 expression in patients with bone (upper row, prostate cancer, red and green arrows) and soft tissue (bottom row, breast cancer) metastases using mAb [^89^Zr]Zr-GSK2849330 [251]. Pre-scan images using bone scan SPECT (top) and ^18^F-FDG (bottom) are given for comparison. These images were originally published in JNM [251]. © SNMMI.

Three clinical studies on HER3 imaging agents have been reported to date. [^64^Cu]Cu-patritumab (also designated as AMG-888 or U3-1287) was the first clinically studied tracer for imaging of HER3 expression after showing good feasibility in preclinical murine models [249,250]. While the imaging procedure and tracer were deemed safe, the imaging contrast (best possible contrast achieved 24 h pi) was only modest due to the low uptake in HER3 expressing lesions. The trial was terminated after the imaging of 11 patients [249]. ^89^Zr-labeled antibodies GSK 2849330 and lumretuzumab allowed for a later scan time, showing optimal imaging contrast 4–7 d pi [73,251]. Both agents could visualize HER3 expression. [^89^Zr]Zr-lumretuzumab detected HER3 expression in 19 of 20 patients and 67.6% of the lesions larger than 10 mm; just over half of them had quantifiable uptake [73]. [^89^Zr]Zr-GSK 2849330 showed a tumor uptake dependent on the injected mass, thus indicating good potential for assessment of target occupancy, and as a tool for dose selection [251]. Figure 5 shows patient images using anti-HER3 mAb [^89^Zr]Zr-GSK 2849330. Saturable tumor uptake was not achieved by injecting the maximum dose of [^89^Zr]Zr-lumretuzumab [73], which might be because it targets a different epitope of the HER3 ECD. The study with [^89^Zr]Zr-lumretuzumab was the only clinical study that includes patients with liver metastases [73]. Unfortunately, none of these lesions could be visualized with positive contrast. This might not be particularly surprising considering the endogenous HER3 expression in liver. The uptake in healthy liver exceeded even the uptake in non-hepatic metastases. Similar problems with hepatic metastases could be expected also for [^64^Cu]Cu-patritumab and [^89^Zr]Zr-GSK 2849330, even though no data are available [249,251]. Lockhart et al. reported a tumor-to-liver ratio below 0.8 for [^64^Cu]Cu-patritumab [249]. In preclinical studies, [^89^Zr]Zr-GSK 2849330 had the highest uptake in the liver, but this mAb is also the only variant that has cross-reactivity for murine ErbB3 [252,253,254]. In addition, both GSK2849330 and lumretuzumab are glycosylated antibodies, which should increase the mAb mediated cytotoxicity (ADCC), but also tends to increase the accumulation in liver due to faster clearance [73,251].

It is worth noting that while the antibodies under clinical investigations have, so far, not shown exceptionally compelling performances as imaging agents for HER3, other antibody-based tracers in preclinical stages have been reported [254,255]. Among these, [^89^Zr]Zr-mab3481 outperformed others, showing a very high accumulation in xenografts (47–52%ID/g) along with high- and dose-dependent tumor-to-blood ratios [255]. The murine origin of mab3481, together with the selected murine model could be the cause, since the selected model is able to produce its own antibodies, which could contribute to saturation of receptors in organs with natural expression. It should furthermore be mentioned that thus far, no humanized version of this mAb is available.

Low levels of overexpression obviously narrow the margin between what are considered “high”, and none- or low expressing tumors, which complicates accurate quantification. None of the clinically studied mAbs could differentiate between HER3 expression levels or reliably quantify all detected lesions [73,249,251]. Moreover, 32% of lesions >10 mm detected with [^89^Zr]Zr-lumretuzumab were falsely identified as HER3-positive [73]. [^89^Zr]Zr-GSK 2849330 merely showed a tendency for a correlation between tumor uptake and receptor expression [251]. This is in direct contrast to preclinical data, where lumretuzumab PET-uptake was well correlated with HER3 expression and [^89^Zr]Zr-GSK 2849330 was able to clearly differentiate between HER3 expressing and non-expressing xenografts [252,253]. The EPR effect in tumors might overshadow the actual HER3 mediated tumor uptake and could lead to such an overestimation. The heterogeneous expression in actual lesions in patients compared with xenograft models is another reason that could explain the discrepancy between preclinical and clinical data. In addition, dynamic and potentially rapidly changing expression also complicates the correlation between uptake and biopsy material. Thus, the long waiting time for sufficient imaging contrast associated with full-length mAb tracers could be disadvantageous and smaller tracers might be better suited to detect early and rapid changes in HER3 expression. A mAb105 derived F(ab′)_2_ fragment ([^64^Cu]Cu-DOTA-mAb105-F(ab′)_2_) was able to visualize changes in response to AKT and PI3K inhibitors 24 h pi (tracer injected 48 h after treatment start) [133]. The uptake of affibody molecules correlated with HER3 expression [256] in preclinical in vivo models, and they were able to monitor receptor occupancy and receptor expression during MM121 and HSP90 therapy [257,258].

### 5.2. sdAb, Affibody Molecules and Peptides for Imaging of HER3 Expression

**Figure 6:**
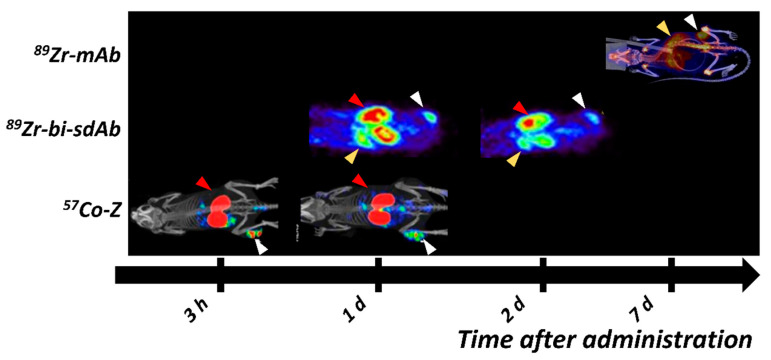
Imaging of HER3 expression (white arrows) in xenografted mice using ^89^Zr-labeled mAb [252], biparatopic dimer of sdAb [259], and ^57^Co-labeled affibody molecule (^57^Co-Z) [265]. Yellow arrows point to the uptake in the liver and red arrows in the kidneys. These images were originally published in J Nucl Med [259] © SNMMI; PlosOne [252] and Int J Mol Sci [265].

An interesting construct, [^89^Zr]Zr-MSB0010853, was proposed by Warnders et al. in the form of a biparatopic nanobody conjugate, consisting of two HER3-targeting sdAbs linked by a third albumin targeting sdAb [259]. The maximum uptake of [^89^Zr]Zr-MSB0010853 in H441xenografts 24 h pi (6.2 %ID/g) was comparable with that of the HER3-targeting affibody molecule Z_HER3:08698_, but lower than for radiolabeled anti-HER3 antibodies reported in preclinical studies. PET images obtained using [^89^Zr]Zr-MSB0010853 together with images obtained using [^89^Zr]Zr-GSK2849330, and a radiocobalt-labeled affibody are presented in Figure 6. The tumor-to-blood ratio for the sdAb-conjugate 96 h pi (33 ± 8) was much higher compared with ratios for the bispecific trastuzumab-Fab-heregulin conjugate 48 h pi (6.3) [260], [^89^Zr]Zr-lumretuzumab 144 h pi (6.6)[253] and [^89^Zr]Zr-GSK2849330 48-144 h pi (~4 [252] ), and most affibody conjugates 3–24 h pi (5–19) [256,261,262,263,264]. A copper-64 labeled F(ab’)_2_ fragment (derived from HER3 mAb 105) showed potential for the imaging of therapy induced changes in HER3 expression in xenograft-bearing mice. However, no full biodistribution of the tracer was reported, making comparison with other tracers difficult [133].

Orlova et al. in 2014 reported the proof-of-principle study of SPECT imaging of HER3 expression using the first generation of ^99m^Tc-labeled anti-HER3 affibody molecules [262]. In this pre-clinical study, tumor-to-blood ratios for [^99m^Tc]Tc-(HE)_3_-Z_HER3:08699_ (6–7) at 4 h pi were already in the same range as for [^89^Zr]Zr-lumretuzumab 6 d pi [253] and [^89^Zr]Zr-GSK2849330 2–4 d pi [252], and higher than for [^89^Zr]Zr-Mab#58 6 d pi [254]. [^18^F]AlF-Z_HER3:08698_ showed that clear visualization of HER3 expressing xenografts is possible, as early as 1 h pi [266]. However, later time points generally provided improved imaging contrast [261,264,265]. Although no complete dose finding study was reported, injected doses between 1–3 µg have been used for imaging purposes and an injected mass of 70 µg resulted in a significant decrease in uptake in xenografts and even in the liver [256,258]. One study showed that the hepatic uptake of an affibody trimer was 10-fold higher compared with the uptake of the monomer [267]. At the same time, the tumor uptake of the monomer and trimer were on the same level, presumably due to the slower extravasation and penetration of the trimer. Co-injection of unlabeled trimer and radiolabeled monomer thus lowered the liver uptake of the monomer and significantly improved the contrast [267]. 

It was further demonstrated that different labels influence the imaging properties of Z_HER3_ in different ways. Several studies have focused on reducing hepatic uptake of anti-HER3 affibody molecules by investigating the influence of molecular design and a radiolabeling approach [89,265,268]. For example, an increased negative charge of the radiometal-chelator complex reduced the hepatic uptake by 2-fold, significantly increasing the tumor-to-liver ratio [89]. Conjugation of a N-terminal (HE)_3_-tag facilitated blood clearance of ^68^Ga-labeled Z_HER3:08698_. Implementation of this molecular design in combination with a NOGADA chelator at C-terminus significantly improved the tumor-to-liver ratio [268]. Among the radiometal labels, radiocobalt labeled (HE)_3_-Z_HER3:08698_-DOTA has shown to be the most promising variant, for the first time exceeding a tumor-to-liver ratio of one, a prerequisite for the detection of HER3 expressing metastases in liver [265]. The use of non-residualizing iodine labeled further increased this ratio to 2.4 (4 h pi), a similar ratio to what was achieved by co-injection of the affibody trimer [107,267]. However, the tumor uptake of [^125^I]I-PIB-(HE)_3_-Z_HER3:08698_-DOTAGA was three times lower than the tumor uptake of [^111^In]In-(HE)_3_-Z_HER3:08698_-DOTAGA and decreased appreciably from 4 to 24 h [107]. 

To our knowledge, only one peptide-based tracer for the imaging of HER3 has been reported. The undecapeptide entitled HER3P1 labeled with ^68^Ga could visualize HER3 expression in murine models, but because of the rapid washout and low affinity, tumor uptake did not exceed 1% ID/g [269]. Optimization, particularly an increased affinity, would be required for it to join affibody molecules and sdAbs as promising imaging probes for HER3 expression. 

### 5.3. HER3 Concluding Remarks 

Table 3 provides a summary over the tracers for PET and SPECT imaging that were discussed above. The low level of HER3 expression in target tissue, the extremely dynamic expression, and natural expression in healthy tissue (e.g., liver) remain the main challenges for good HER3 imaging contrast. Proposed antibody-based tracers are modifications of pre-existing molecules, first and foremost developed for therapy. Their only m oderate success in imaging clinical trials shows that this approach might not be sufficient for the imaging of such a particularly challenging target as HER3. With the increasing need for the assessment of HER3 status in patients with various malignancies, more alternatives to mAbs need to be studied in a clinical setting. Tracers based on sdAb and affibody molecules designed specifically for PET and SPECT imaging show extremely promising results and should be explored in translational studies. 

## 6. Conclusions

The EGFR (RTK I) family takes a central role in oncogenic transformation. The movement towards precision medicine and advances in treatment regimens, including EGFR-family targeting therapeutics, increase the need for non-invasive repeatable methods for the detection of receptor expressions. In recent years, PET and SPECT imaging have been recognized as useful and powerful alternatives to traditional methods for molecular profiling. They can be applied throughout the treatment course: from whole-body target detection and quantification of target expression, therapy planning and dose finding to the monitoring of treatment response. Together with the optimal tracers, PET and SPECT can thus be key assets for more efficient cancer treatment with EGFR-family targeted drugs.

Antibodies have led the way in the development of imaging agents against receptors of the EGFR family. However, the data compiled in this review shows a noticeable trend of moving away from tracers based on mAbs and their derivatives toward engineered antibody fragments, mainly sdAb (nanobodies), and ESPs (affibody molecules, ADAPT, DARPins).

The development of HER2-targeting imaging agents is still the furthest advanced with several novel alternatives to antibody-based tracers, e.g., sdAb, affibody molecules, ADAPTs, and DARPins in clinical trials. HER1 and HER3 are more challenging targets by nature, but nonetheless, tracers against both targets have made the jump to clinical investigation. Based on the pioneering work on HER2, the lessons learned hopefully can facilitate and accelerate tracer development against other family members in the future. Once the role of HER4 in malignant transformations becomes clearer, and the development of potential HER4 targeted therapeutics intensifies, the pre-existing knowledge of HER-targeting diagnostic agents can expedite the development of HER4 diagnostic agents.

The diversity of PET and SPECT tracers for imaging of RTK Class I presented here is encouraging. Some probes can be used for molecular profiling of tumors with high contrast within few hours after administration (ESPs and mAb fragments). Others could be used to estimate receptors accessibility before therapy (radiolabeled therapeutic mAbs), and third ones can be used for monitoring therapy response and receptor occupancy under treatment (probes targeting receptor’s epitope different with therapeutic mAb). It would be desirable to create a “toolbox” with a well-rounded selection of different targeting molecules and radiolabeling methods which allow for choosing the best tracer to answer the medical question at hand.

## Figures and Tables

**Table 1:** Overview of the most promising HER1-targeting Positron Emission Tomography (PET) and Single Photon Emission Tomography (SPECT) tracers in clinical and preclinical development.

Tracer	PET/ SPECT	Type of Molecule	Key Points	Reference
Clinical Studies				
[^89^Zr]Zr-cetuximab	PET	Antibody	Phase II/II Specific tumor uptake in patients Optimal imaging day six	[75,111,130]
[^89^Zr]Zr-panitumumab	PET	Antibody	Tumors could not be visualized, potentially be due to location in areas with high background due metabolic tracer accumulation (liver, abdomen)	[81]
[^89^Zr]Zr-nimotuzumab	PET	Antibody	Currently in Phase I/II studies Estimated absorbed was lower than dose for [^89^Zr]Zr-DFO-panitumumab	[123], NCT04235114
Preclinical Studies				
[^64^Cu]Cu-cetuximab	PET	Antibody	Can visualize HER1 expression 48 h pi xenografts Correlation between uptake, expression and predicative power still unclear	[114,115]
[^64^Cu]Cu-panitumumab	PET	Antibody	HER1 expression in three different HNSCC xenografts could be visualized Inverse relation between tumor uptake and HER1 expression, possibly related to vessel density, vascular permeability and binding site barrier	[128]
[^111^In]In-panitumumab	SPECT	Antibody	[^111^In]In-CHX”-DTPA- panitumumab has slightly faster blood clearance compared with [^89^Zr]Zr-DFO-panitumumab Conjugation of an MCP with 13 DOTAs to reduce liver and spleen uptake surprisingly increased hepatic uptake significantly	[121]
[^64^Cu]Cu-NOTA- panitumumab F(ab′)_2_	PET	Antibody fragment	Faster clearance compared with full length panitumumab Highest uptake in xenografts 48 h pi Tumor-to-blood ratios of 5–9 at 48 h pi	[131,161]
[^111^In]In-cetuximab F(ab′)_2_	SPECT	Antibody fragment	Provides significantly better tumor-to-blood ratios than cetuximab at early time points Highest uptake in xenografts 24 h pi Correlation between uptake and expression in xenografts Therapy response in mice correlated with tracer uptake	[87,132,162]
[^99m^Tc]Tc-Pm-Fab-His_6_	SPECT	Antibody fragment	Tumor uptake and tumor-to-blood ratios similar to those of [^64^Cu]Cu-NOTA-panitumumab-F(ab′)_2_ 24 h and 48 h pi Clear visualization of a panel of HER1 expressing xenografts using SPECT	[134]
[^99m^Tc]Tc-D10	SPECT	sdAb	Detection of HER1 expressing lesions 45 min pi Tumor-to-blood ratio comparable to that of F(ab′)_2_ fragments of cetuximab and panitumumab	[135]
[^68^Ga]Ga/[^89^Zr]Zr-Df-Bz-NCS-7D12	PET	sdAb	Higher tumor uptake than uptake of [^ 99m ^ Tc]Tc-D10 Good visualization on PET 1h pi	[137]
[^89^Zr]Zr-DFO-Z_EGFR:2377_	PET	Affibody	Tumor uptake 3 h pi exceeded uptake of [^89^ Zr]Zr-DFO-cetuximab at 48 h pi Tumor-to-tissue contrast at 3 h and 24 h was higher than [^89^ Zr]Zr-DFO-cetuximab except from tumor-to-kidney	[140]
[^55/57^Co]Co-DOTA- Z_EGFR:2377_	PET	Affibody	Highest tumor-to-tissue contrast compared with all other studied affibody-based tracers	[105]
[^68^Ga]Ga-DFO-Z_EGFR:2377_	PET	Affibody	For imaging at 3 h pi Compared with other affibody molecules against HER1 the imaging contrast was inferior only to the radiocobalt conjugate, but ^68^Ga is more available than ^55^Co	[142]
[^64^Cu]Cu-Fn_EI3.4.3′_	PET	Fibronectin binding domain	Good tumor visualization on PET 1h pi No cross-reactivity with mErbB1	[149]

**Table 2:** Overview of the tracers undergoing clinical trials for PET and SPECT imaging of HER2 expression and most promising tracer under preclinical development.

Tracer	PET/ SPECT	Type of Molecule	Key Points	Reference
Clinical Studies				
[^64^Cu]Cu-trastuzumab [^89^Zr]Zr-trastuzumab	PET	Antibody	[^89^Zr]Zr-trastuzumab was the first clinically studied PET-tracer for imaging of HER2 Successful visualization of HER2 positive lesions in patients with BC and esophagogastric cancer Optimal imaging 4–6 d pi (^89^Zr) or 48 h pi (^64^Cu)	[74,179,180,181,185,187]
[^89^Zr]Zr-pertuzumab	PET	Antibody	Successful visualization of HER2 positive primary tumor and metastases in BC patients [^89^Zr]Zr-pertuzumab appeared to have slightly higher uptake than [^89^Zr]Zr-trastuzumab in liver, kidney, spleen, and lung	[182,184]
[^111^In]In-CHX-A″-DTPA trastuzumab	SPECT	Antibody	Phase 0 demonstrated safety No data on tumor targeting available	[177]
[^68^Ga]Ga-DOTA-F(ab′)_2_- trastuzumab	PET	Antibody fragment	HER2-positive lesion were detected in only half the patients with known HER2-positive disease In xenografted mice, the tumor uptake was proportional to HER2 expression	[206,209]
[^68^Ga]Ga-HER2-Nanobody (also 2Rs15d)	PET	sdAb	Well tolerated administration Imaging of HER2 expression 60–90 min pi 90 min pi was suggested as more suitable time point for imaging	[223]
[^131^I]I-GMIB-Anti-HER2- VHH1 (also GMIB-2Rs15d)	SPECT	sdAb	Phase I trial showed uptake in patients with lesions >3 cm Visualization of smaller lesions was difficult, supposedly due to PVE	[219]
[^68^Ga]Ga-DOTA-ABY025	PET	Affibody	Primary tumors and metastases (even hepatic) could be visualized Correlation between SUV in lesions with confirmed HER2 IHC scores Multicenter Phase II/III study currently underway (NCT03655353)	[78,233]
[^68^Ga]Ga-NOTA-MAL-Cys-MZHER2:342	PET	Affibody	Optimal imaging contrast was achieved 2 h pi Reporting 100% specificity, 55% sensitivity at an SUV cutoff at 6.6	[237]
[^99m^Tc]Tc-ADAPT6	SPECT	ADAPT	First ever clinical study with ADAPT Well-tolerated administration An optimal injected protein dose (500 µg) was able to differentiate between positive and negative lesions	[169]
[^99m^Tc]Tc-(HE)_3_-G3	SPECT	DARPin	First clinical trial with HER2 targeting DARPin to assess safety, distribution, and dosimetry in patients with primary breast cancer is currently ongoing	NCT04277338
Preclinical Studies				
[^64^Cu]Cu-NOTA-pertuzumab F(ab′)_2_	PET	Antibody fragment	Trastuzumab-induced changes in HER2 could be detected Sensitivity increased from 24 to 48 h pi	[205]
[^89^Zr]Zr-Df-Fab-PAS200 [^124^I]I-Df-Fab-PAS200	PET	Antibody fragment	Despite lower tumor uptake of the ^124^I-labeled variant both tracers showed comparable contrast and good visualization of xenografts 24 h pi Administration of ^89^Zr-labeled variant in one patient could detect lesions 24 h pi	[207,208]
[^123/124/125^I]I-PIB-G3-(HE)_3_	PET/ SPECT	DARPin	Indirect iodination using SPIB and introduction of (HE)_3_-tag improved tumor-to-tissue ratios Another promising variant for clinical translation among the explored DARPin variants	[86,224,239]
[^111^In]In-DTPA-AHNP-PEG	SPECT	Peptide	Showed higher tumor uptake, retention and tumor-to-tissue ratios than other reported HER2 targeting peptides Good visualization of gastric cancer xenografts up to 48 h pi	[242]

**Table 3:** Overview of the HER3-targeting imaging agents for PET and SPECT.

Tracer	PET/ SPECT	Type of Molecule	Key Points	Reference
Clinical Studies				
[^64^Cu]Cu-patritumab (also AMG-888 or U3-1287)	PET	Antibody	Clinical/Phase 1 terminated after 11 patients due to low uptake in HER3 expressing lesions	[249]
[^89^Zr]Zr-GSK 2849330	PET	Antibody	Tumor uptake was dependent on the injected mass thus indicating good potential for assessment of target occupancy and as a tool for dose selection	[251]
[^89^Zr]Zr-lumretuzumab (also RG-7116)	PET	Antibody	Detected 67.6% of lesions larger than 10 mm No liver metastases could be detected due to high uptake in healthy liver tissue	[73]
Preclinical Studies				
[^89^Zr]Zr-MSB0010853	PET	Biparatopic sdAb construct	Maximum tumor uptake 96 h pi was comparable with that of affibody molecules 3–24 h piHigher tumor-to-blood ratios compared with preclinical data on [^89^Zr]Zr-lumretuzumab and [^64^Cu]Cu-patritumab	[259]
[^55/57^Co]Co-(HE)_3_- Z_HER3:08698_-DOTA	PET/ SPECT	Affibody	Reported best tumor-to-liver contrast among radiometal labeled anti-HER3 affibody molecules	[265]
[^89^Zr]Zr-DFO-Z_HER3:08698_	PET	Affibody	Could image changes in HER3 expression induced by HSP90 therapy	[258]
[^68^Ga]Ga-HER3P1	PET	Peptide	Uptake in xenografts <1 %ID/g due to rapid wash out and low affinity	[269]

## Abbreviations

BC	Breast cancer
ECD	Extra cellular domain
EGFR	Epidermal growth factor receptor
ESP	Engineered scaffold proteins
HER	Human epidermal growth factor
ICD	Intra cellular domain
IHC	Immunohistochemistry
ISH	in situ hybridizations
mAb	Monoclonal antibody
mBC	Metastatic breast cancer
mCRC	Metastatic colorectal cancer
NSCLC	Non-small cell lung cancer
PC	Prostate cancer
PET	Positron emission Tomography
pi	post injection
RTK	Receptor tyrosine kinase
scFv	Single chain variable fragment
sdAb	Single domain antibody
SPECT	Single photon emission tomography
